# Enhancing the Accuracy of Platelet to Lymphocyte Ratio after Adjustment for Large Platelet Count: A Pilot Study in Breast Cancer Patients

**DOI:** 10.1155/2012/653608

**Published:** 2012-12-13

**Authors:** Charalampos Seretis, Fotios Seretis, Emmanuel Lagoudianakis, Marianna Politou, George Gemenetzis, Nikolaos S. Salemis

**Affiliations:** ^1^2nd Department of Surgery, Breast Unit, 401 General Army Hospital of Athens, Kanellopoulou and Katehaki Avenue, 11525 Athens, Greece; ^2^Department of Hematology, Areteion University Hospital, Medical School of Athens, Greece

## Abstract

*Background*. The objective of our study is to investigate the potential effect of adjusting preoperative platelet to lymphocyte ratio, an emerging biomarker of survival in cancer patients, for the fraction of large platelets. *Methods*. A total of 79 patients with breast neoplasias, 44 with fibroadenomas, and 35 with invasive ductal carcinoma were included in the study. Both conventional platelet to lymphocyte ratio (PLR) and the adjusted marker, large platelet to lymphocyte ratio (LPLR), were correlated with laboratory and histopathological parameters of the study sample. *Results*. LPLR elevation was significantly correlated with the presence of malignancy, advanced tumor stage, metastatic spread in the axillary nodes and HER2/neu overexpression, while PLR was only correlated with the number of infiltrated lymph nodes. *Conclusions*. This is the first study evaluating the effect of adjustment for large platelet count on improving PLR accuracy, when correlated with the basic independent markers of survival in a sample of breast cancer patients. Further studies are needed in order to assess the possibility of applying our adjustment as standard in terms of predicting survival rates in cancer.

## 1. Introduction

The close interplay between inflammation, coagulation, and cancer progression has become a field of intense scientific research [1]. While the exact pathophysiological mechanisms that rule the formation of vicious circles among coagulation parameters, inflammatory indices and tumor cells still remain unclear, there is a growing interest for clinical interpretation of these interactions, resulting in the establishment of novel biomarkers in surgical oncology [2]. These emerging biomarkers try to combine the evident preinflammatory and precoagulative status in cancer with the endogenous residual anticancer capability; neutrophil to lymphocyte and platelet to lymphocyte ratio (NLR and PLR) are, respectively, these novel biomarkers, being both reliable and cost-effective [3]. Although the credibility of NLR [4–6] as a prognostic marker in solid cancers has been widely accepted, PLR has failed to be considered as an equally undisputed prognostic biomarker since, in contrast to the existence of solid data coming from studies assessing the prognostic significance of NLR, there are many studies that question the equal accuracy of PLR [7–10]. 

From a more pathological point of view, platelet activation is proven to be of paramount importance concerning the progression of malignancy. Recent experimental and clinical data suggest that the activation of platelets is a hallmark in the natural course of cancer, by promoting neoangiogenesis, degradation of the extracellular matrix, release of adhesion molecules, and growth factors, all of which are essential components for further tumor growth and metastatic spread [11–13]. Apart from this negative oncological impact that platelet activation has in cancer, various studies clearly demonstrate that, in many types of cancer, the release of proinflammatory cytokines, such as IL-1, IL-3, and IL-6, clearly promotes megakaryocytes' proliferation, resulting in the gradual establishment of thrombocytosis [14, 15]. This exact stimulation inevitably leads to an increased detection of more primitive types of circulating platelets [16]. Thus, it is absolutely justified to claim that the evaluation of platelet count and functional status is consistent with the progression of malignancy [17, 18]. 

In terms of laboratory, these two elements, platelet activation, and response to platelet overconsumption, are combined in an overlooked parameter, the platelet-large cell ratio. Calculated by the majority of modern automated hematological analysts, platelet-large cell ratio is defined as the percentage of platelets that exceed the normal value of platelet volume (in our study 12 fl or more) [19]. Multiplying by the total platelet count, we can easily assess the large platelet count in any blood sample. In particular, the importance of measuring the amount of circulating large platelets in cancer patients lies upon two facts: firstly, activated platelets, by increasing their volume, they become rounder and expose their surface receptors, which is a crucial initial step for their aggregation and adhesion [20, 21]. Secondly, increased platelet-large cell ratio is definitely suggestive of an increased release of platelets from the bone marrow as a response to peripheral proliferative signals, as exactly happens in cancer, through the megakaryocyte-proliferative effect of the malignant cells [16]. The increased fraction of these more immature platelets is morphologically closer to the more primitive type of the megakaryocyte. Although more primitive, these immature platelets are enzymatically more active than the smaller ones, having more granules and adhesion receptors, which are considered of paramount importance in cancer progression and metastasis [22, 23]. 

As far as PLR is concerned, considering that the number of circulating lymphocytes is an undisputed prognostic marker in surgical oncology, reflecting to a great extent the endogenous anticancer capability of the immune system [24, 25], it would be very challenging to examine if the adjustment of conventional PLR for the count of large platelets would improve the accuracy of this emerging—but also disputed—biomarker, providing a potentially possible explanation for the controversial results of the existing studies, which assessed the prognostic significance of PRL elevation in patients with solid cancers.

## 2. Patients and Methods

To test our hypothesis, we performed a cross-sectional study, in which 79 women with neoplastic lesions of the breast were enrolled, who were surgically treated in our Department. More specifically, our study sample constituted of 44 patients with fibroadenomas (group A), who underwent local excision, and 35 patients with unifocal breast cancer, submitted to modified radical mastectomy. Concerning the breast cancer group, in order to standardize the oncological profile of the tumors, only patients with unifocal grade II (*n* = 18, group B) and III (*n* = 17, group C) invasive ductal carcinoma were included. The rest of the other exclusion criteria were the presence of hematological disorders, active inflammation, anemia, and recent venous thrombosis (past 6 months). Aiming to test our hypothesis in a sample of patients as wider as possible, we did not exclude patients under antiplatelet or anticoagulation therapy. As both antiplatelet and anticoagulants would be expected to contribute to a less active precoagulative and thrombotic status, in case of confirmation of our study hypothesis, it would be reasonable to assume that in patients under no such medication our proposed adjustment would be even more favorable in terms of improving the accuracy of “conventional” PLR. At this point, it should be mentioned that we decided to assess this adjustment in breast cancer patients deliberately, for exactly the same reason we described above; breast cancer is considered to be one of the less precoagulative types of solid cancer [26, 27] and possible confirmation of our assumption in a less precoagulative type of malignancy would also apply in more thrombotic cancers, as the gastrointestinal ones. 

“Conventional” PLR was calculated as the ratio of total count of platelets, divided by the total count of lymphocytes, in fasting morning blood samples, obtained from our patients during the process of routine preoperative general blood testing. The adjusted PLR for large platelets (LPLR) was calculated after multiplying the “conventional” PLR with the platelet-large cell ratio, as calculated by the automatic analyst in the general blood test result. The blood samples were obtained in the morning before the scheduled operation, between 07.30 and 09.00, in order to standardize the known impact of circulating hormones (circadian rhythm) on the number and subtype distribution of the various white blood cell indices. Moreover, the blood samples obtained were fasting, in accordance with the Department's protocol for routine preoperative evaluation of the patients scheduled for elective surgery, which is applied for the standardization of the preoperative values of biochemical tests.

In order to assess the correlation of both PLR and LPLR with the extent of fibrinolysis, we obtained blood samples to measure the level of circulating d-dimers in 16 patients with fibroadenomas (group A) and 25 patients with breast cancer (12 from group B, 13 from group C). 

We evaluated the existence of differences in PLR and LPLR values regarding the benign or malignant nature of the tumor (group A versus groups B and C), the histological grade, the number of infiltrated axillary lymph nodes, the infiltrated/removed lymph node ratio, and the size of the primary tumor. Also, we correlated PLR and LPLR with d-dimers, marker suggestive of active fibrinolysis and also emerging biomarker of aggressive types of breast cancer [28, 29], as well as with the neutrophil to lymphocyte ratio (NLR), which is independent prognostic factor of survival in various types of cancer and breast cancer included [4–6]. At last, due to the relative small sample of our cancer patients and the considerable number of inconclusive histopathological reports regarding the overexpression or absence of hormonal receptors in the resected specimens, we did not correlate the abovementioned laboratory parameters with immunohistochemical data. However, we assessed potential differences with respect to the overexpression of HER-2-neu status.

Our statistical analyses were performed using SPSS 16.0 software package. All variables were assessed for the values' normal distribution. Correlations of PLR and LPLR with the evaluated parameters were performed using *t*-test and one-way ANOVA test. A *P* < 0.05 level was considered statistically significant.

## 3. Results

**Figure 1:**
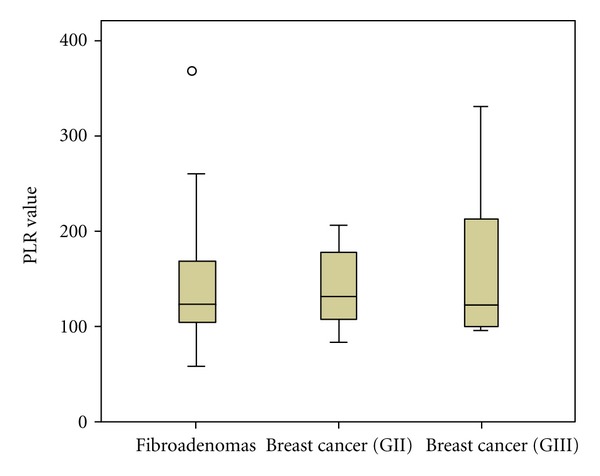
PLR values within groups A, B, and C (fibroadenomas, invasive ductal carcinoma grade II and III, resp.).

**Figure 2:**
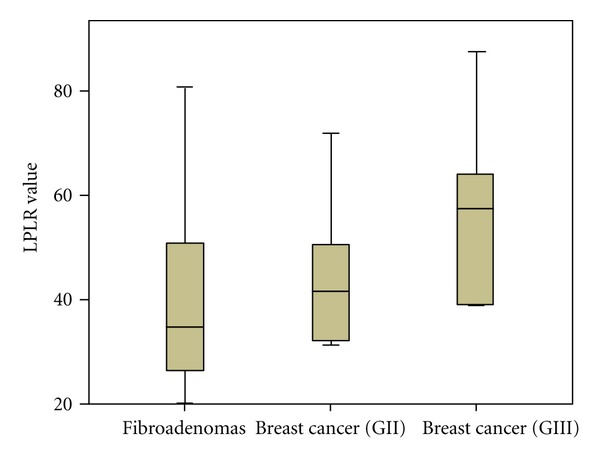
LPLR values within groups A, B, C (fibroadenomas, invasive ductal carcinoma grade II and III, resp.).

The baseline demographic and laboratory characteristics of the three groups (A, B, and C) are demonstrated in Table 1. Overall, our analysis revealed that LPLR, NLR and d-dimers were significantly elevated in patients with breast cancer, compared to patients with fibroadenomas. No statistical significant differences occurred for the values of platelet-large cell ratio (*P* = 0.091), as well as, interestingly, PLR was not significantly elevated when comparing the three groups of patients, without even being close to the level of statistical significance (*P* = 0.558). The differences of our laboratory parameters within the groups are presented in Table 2. LPRL was significantly elevated comparing groups A and C (fibroadenomas and grade III IDC), as exactly were NLR and d-dimers. Nevertheless, no statistically significant differences occurred when comparing LPRL, as well as NLR, d-dimers and platelet-large cell ratio between groups A and B or B and C. The enhanced accuracy of PLR after the adjustment for the fraction of large platelets (LPLR) is visualized in Figures 1 and 2. 

**Figure 3:**
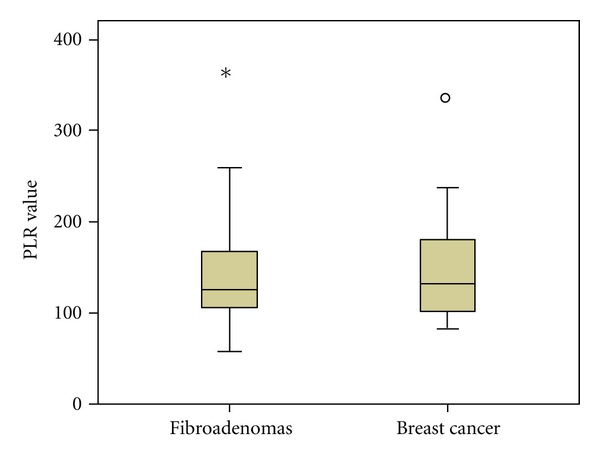
PLR values in groups A versus B and C (fibroadenomas versus breast cancer).

**Figure 4:**
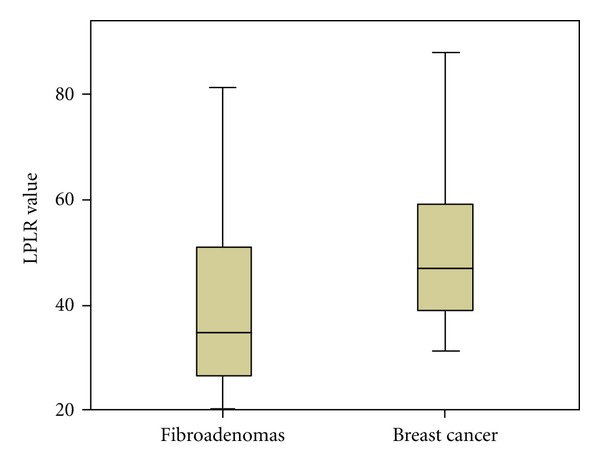
LPLR values in groups A versus B and C (fibroadenomas versus breast cancer).

As PLR values were not significantly different when comparing groups A, B and C separately, we examined the existence of any potential differences in PLR values between the patients with benign neoplasia (group A) and breast cancer in general (groups B and C together). Again, as presented in Figures 3 and 4, PLR elevation was not significant (*P* = 0.9), in contrast with LPLR elevation (*P* = 0.028). Similarly to our previous findings, only NLR and d-dimers were significantly elevated in patients with breast cancer (group A versus groups B and C together, *P* < 0.001 for both NLR and d-dimers), while no significant differences occurred concerning the values of platelet-large cell ratio.

**Figure 5:**
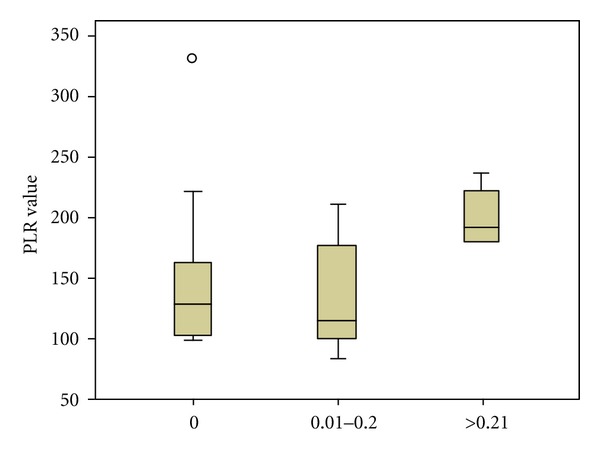
PLR values according to infiltrated/retrieved lymph node ratio value.

**Figure 6:**
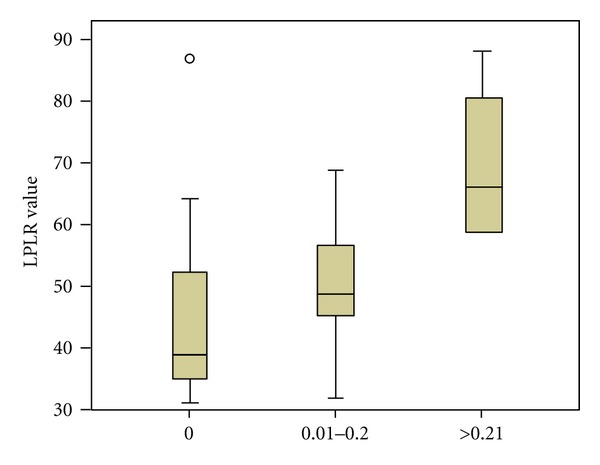
LPLR values according to infiltrated/retrieved lymph node ratio value.

With respect to the extent of lymphatic spread in the groups B and C, in absolute numbers, PLR, LPRL, d-dimers, NLR, and platelet-large cell ratio were all significantly correlated with a higher number of infiltrated lymph nodes and a higher infiltrated-to-retrieved lymph node ratio (*P* < 0.05 for all the parameters). However, when we categorized the breast cancer patients in three groups, according to the value of infiltrated-to-retrieved lymph node ratio, aligned with the cut-off ratios of previously published analyses [30] (0, 0.01–0.2, >0.21 with *n* = 16, 14, and 5 patients in each subgroup), only elevated LPLR and d-dimers (and surprisingly not NLR) were statistically significantly elevated in the patients with extended lymphatic spread in the axillary nodes (*P* = 0.01 and *P* = 0.037 resp., PLR and LPLR compared in Figures 5 and 6).

Concerning the histopathological characteristics of the breast cancer patients, elevated NLR, PLR, LPLR, d-dimers, and platelet-large cell ratio were significantly correlated with larger tumor size (*P* < 0.05 for all the parameters), while, regarding the positivity for HER2/neu overexpression, only LPLR and d-dimers were statistically significantly increased in the group of patients with positive staining (*n* = 8), compared to the patients with absence or moderate expression of HER2/neu (*n* = 27). At last, it should be mentioned that LPLR, PLR, platelet-large cell ratio, NLR, and d-dimers values were statistically significantly correlated with each other, in every group (A, B, and C) separately and in the entire cohort. 

## 4. Discussion

Our study evaluated for the first time the potential benefit of adjusting platelet to lymphocyte ratio (PLR), an emerging prognostic factor of survival in various types of solid cancers, using the fraction of large platelets (large platelet to lymphocyte ratio (LPLR)). Despite the fact that we did not assess directly the effect of our modification on the enhancement of the predictive value of PLR, as we did not correlate it with survival rates, it is evident that LPLR was correlated with all the standard predictors of survival in breast cancer-tumor stage, number of infiltrated lymph nodes, infiltrated/retrieved lymph node ratio, HER2/neu overexpression, and primary tumor size. Although it would be very challenging, our relatively small number of cancer patients and the significant amount of inconclusive histopathological examinations upon the expression of hormonal receptors did not allow any further relative statistical analyses. Moreover, the existence of significant age differences of our patients' groups definitely poses serious limitations with respect to the interpretation of the clinical parameters of primary focus (large platelet percentage, NLR, d-dimers). To tackle this problem, the implementation of a correction factor would have been of great value. In the framework of our study, it was not feasible to describe or propose a potential correction factor for correcting the age-related differences in the laboratory parameters tested, as our primary aim was to attempt a “head-to-head collision” between PLR and LPLR, which were compared using the same patient groups.

As mentioned, the main aim of our study was to report the potential beneficial effect of adjusting platelet to lymphocyte ratio (PLR) for large platelets (LPLR), correlating the conventional and the modified biomarker with the standard independent prognostic factors in breast cancer. Apart from this, we assessed the presence of any significant differences of PLR and LPLR between patients with unifocal malignant and benign lesions. Our rationale behind the latter was that LPLR could be of potential value in the differential diagnosis of breast lesions, possibly in the framework of multi-factorial prognostic scores, as well as a parameter of identifying high-risk patients in cases of borderline malignancies. Herein, from this point of view, we reported that LPLR is superior to the conventional PRL, which did not differ significantly between the patients with fibroadenomas and breast cancer, respectively. 

It became profound that the fraction of large platelets may play a key role in the process of malignancy. However, changes in platelet sizes are not uncommon laboratory findings. These changes can occur during the natural course of various hematological disorders, active inflammation, anemia, and recent venous thrombosis (which were set as exclusion criteria in our study), as well obesity, diabetes mellitus, dyslipidemia, hypertension, and generally conditions characterized by impaired cardiometabolic function [31, 32]. Thus, it would be very reasonable to assume that an elevation of the proportion of large platelets would be expected in any case in some of our patients, and, consequently, these changes might not be associated with more active types of malignancy. However, there are many studies stating that all of the abovementioned conditions stand as risk factors for the development of various types of cancers [33, 34], breast cancer included, indicating that platelet activation and/or thrombocytosis that characterize these conditions is a possible link between them. In other words, these comorbidity factors are in fact contributing towards the progression of malignancy, since they promote a thrombotic profile (via platelet activation and/or thrombocytosis), which triggers neoangiogenesis and favors neutrophilia, resulting in overexpression of vascular growth factors and lower lymphocyte count, respectively. Consequently, even if platelet activation and/or thrombocytosis could be attributed solely to the presence of cardiovascular and metabolic diseases, this fact stands on its own as a risk factor for the development and progression of cancer and should under no circumstances be regarded as an exclusion criterion, enabling the inclusion of a large pool of patients in any future similar studies.

As mentioned, the fraction of large platelets consists of two arms, the activated platelets, which, by increasing their volume, are able to expose their receptors for aggregation and adhesion, and a significant number of morphologically immature platelets, which are closer to the primitive form of megakaryocyte. However, this particular morphological immaturity does not result in impaired enzymatically active potential; indeed, these more primitive platelets are more active than the smaller ones, having larger amounts of granules that contain adhesion molecules and growth factors, with the last being currently in the center of anticancer research. Thus, large platelets could be the spearhead of the circulating platelets with respect to platelet-cancer interactions. However, our results cannot highlight a potential use of the platelet-large cell ratio solely as a marker of higher-grade malignancy although an increased value was correlated with lymphatic spread and tumor size, being a clear proof of the crucial role of platelets concerning tumor growth and metastatic activity. To the best of our knowledge, there are no published studies examining the usefulness of platelet-large cell ratio in solid cancers, while the poor prognostic value of thrombocytosis has been evaluated thoroughly [35]. 

Reviewing the literature concerning the value of PLR as a prognostic factor of survival rates in cancer, we realized two facts. At first, there are several studies that do not support the value of PLR as an independent prognostic factor of survival, in contrast with his “twin” marker, NLR, that is widely accepted in the vast majority of relevant studies [4–10]. Secondly, the majority of those studies in favor of PLR predictive accuracy were conducted in pancreatic cancer, which is associated with extremely prothrombotic profile, clinically manifested with high frequency of thrombotic episodes in these patients [36, 37]. Recently, large studies have demonstrated that pancreatic cancer is associated with high thrombotic risk, while breast cancer is one of the less hypercoagulative cancers [26, 27]. Thus, we believe that the prognostic value of PLR, to an extent which cannot be neglected, lies on the prethrombotic profile of the underlying cancer type, and this is the particular reason we tested our hypothesis in the “relatively hypocoagulative” breast cancer.

Summarizing, the aim of this study is to record the existence of an apparently useful adjustment to PLR, using a widely available laboratory platelet parameter, the platelet-large cell ratio. Never before assessed in solid malignancies, the fraction of large platelets, in combination with the number of circulating lymphocytes, seems to be a promising marker of tumor growth and metastatic activity. We strongly believe that our results merit further evaluation in larger studies; nevertheless, based on commonly accepted pathophysiological principles, we presume that they will be applicable not only in breast cancer, but also in other types of solid cancers, providing a simple and cost-effective biomarker of cancer surveillance.

## Figures and Tables

**Table 1:** Display and comparison of the evaluated parameters of our study sample.

Mean values	Group A	Group B	Group C	*P* value
	(fibroadenomas)	(grade II IDC)	(grade III IDC)	
Age (years)	45.5 (23–73)	55.29 (40–85)	65.39 (39–74)	<0.001
PLR (%)	137.94 (57.7–354.17)	145.48 (83.3–206.25)	160.15 (95.5–330.83)	**0.558**
Platelet-large cell ratio (%)	30.87 (18.7–47.2)	33.02 (24–50.2)	35.76 (26.3–49.5)	0.091
LPLR (%)	40.88 (20.38–81.1)	44.27 (31.35–72)	55.91 (38.66–87.77)	**0.005**
NLR (%)	1.77 (0.64–3.31)	2.27 (1.43–4.07)	2.68 (1.64–4.08)	<0.001
d-dimers (ng/mL)	288.22 (176.9–694.3)	586.4 (170.2–870.08)	650.68 (170.2–965.3)	<0.001

**Table 2:** Comparison of the evaluated parameters within Groups A & B, B & C, and A & C.

Values	Group A versus Group B	Group B versus Group C	Group A versus Group C
PLR	0.844	0.485	0.735
LPLR	0.662	0.64	0.008
Platelet-large cell ratio	0.62	0.51	0.514
NLR	0.001	0.209	0.012
d-dimers	<0.001	<0.001	0.690
age	<0.001	0.69	0.035
